# A covalent antagonist for the human adenosine A_2A_ receptor

**DOI:** 10.1007/s11302-016-9549-9

**Published:** 2016-12-03

**Authors:** Xue Yang, Guo Dong, Thomas J.M. Michiels, Eelke B. Lenselink, Laura Heitman, Julien Louvel, Ad P. IJzerman

**Affiliations:** 1 Division of Medicinal Chemistry, Leiden Academic Centre for Drug Research, Leiden University, P.O. Box 9502, 2300 RA Leiden, the Netherlands; 2 Jiangsu Key Laboratory of New Drug Research and Clinical Pharmacy, Xuzhou Medical University, 209 Tongshan Road, Xuzhou, Jiangsu 221004 China

**Keywords:** G protein-coupled receptors, A_2A_ adenosine receptor, Adenosine, Covalent antagonist, Radioligand binding

## Abstract

The structure of the human A_2A_ adenosine receptor has been elucidated by X-ray crystallography with a high affinity non-xanthine antagonist, ZM241385, bound to it. This template molecule served as a starting point for the incorporation of reactive moieties that cause the ligand to covalently bind to the receptor. In particular, we incorporated a fluorosulfonyl moiety onto ZM241385, which yielded LUF7445 (4-((3-((7-amino-2-(furan-2-yl)-[1, 2, 4]triazolo[1,5-*a*][1, 3, 5]triazin-5-yl)amino)propyl)carbamoyl)benzene sulfonyl fluoride). In a radioligand binding assay, LUF7445 acted as a potent antagonist, with an apparent affinity for the hA_2A_ receptor in the nanomolar range. Its apparent affinity increased with longer incubation time, suggesting an increasing level of covalent binding over time. An in silico A_2A_-structure-based docking model was used to study the binding mode of LUF7445. This led us to perform site-directed mutagenesis of the A_2A_ receptor to probe and validate the target lysine amino acid K153 for covalent binding. Meanwhile, a functional assay combined with wash-out experiments was set up to investigate the efficacy of covalent binding of LUF7445. All these experiments led us to conclude LUF7445 is a valuable molecular tool for further investigating covalent interactions at this receptor. It may also serve as a prototype for a therapeutic approach in which a covalent antagonist may be needed to counteract prolonged and persistent presence of the endogenous ligand adenosine.

**Electronic supplementary material:**

The online version of this article (doi:10.1007/s11302-016-9549-9) contains supplementary material, which is available to authorized users.

## Introduction

G protein-coupled receptors (GPCRs), all membrane-bound proteins, represent one of the largest classes of drug targets and are the anchor point for approx. one third of all marketed drugs [1]. These proteins are notoriously difficult to handle outside of their natural membrane context, for instance, in receptor purification and crystallization. Recently, however, a combination of technological advances has allowed the structure elucidation of an increasing number of these important drug targets [2–4]. In this context, covalent modification of the receptor with ligands is emerging as a useful way to investigate ligand-receptor binding domains in membrane proteins, also because such covalent ligands, acting as pharmacological chaperones, tend to stabilize the otherwise fragile receptor proteins.

Covalent binding of both agonists and antagonists to adenosine receptors has known a long history in purinoceptor research. In the 1980s, the adenosine A_1_ receptor was the preeminent target for such studies [5], eventually leading to the design of a covalently binding fluorosulfonyl derivative of the reference antagonist DPCPX, named FSCPX, which appeared useful also in an in vivo setting [6, 7]. Likewise, the adenosine A_2A_ receptor has been subjected to such strategies. One existing example is the *para*-fluorosulfonyl derivative of SCH58261, FSPTP, which was used to investigate the level of adenosine A_2A_ receptor reserve for agonist activity [8]. The hA_2A_AR has relevance in various diseases, and thus, agonists for increasing blood flow during cardiac nuclear stress tests [9] and an antagonist for the treatment of Parkinson’s disease [10] are on the market, and the receptor may also play a role in cancer-immunotherapy [11]. The hA_2A_AR has also been one of the first GPCRs to be crystallized and a wide variety of crystal structures has been published, including the reported structures co-crystalized with agonist UK-432097 or antagonist ZM241385 [12–14]. Although covalent A_2A_R antagonists have been previously synthesized and investigated in terms of their affinity or potency [8, 15–18], little is known about their precise binding mode in the receptor and their effects on the kinetics of interaction.

In this study, we describe our efforts to obtain a covalent antagonist probe for the hA_2A_AR, as a logical extension of our previous research on long residence time antagonists, i.e., compounds that dissociate only slowly from the receptor [19]. We used the antagonist ZM241385 as the starting point in our design efforts and synthesized a fluorosulfonyl derivative of it, LUF7445. We then validated this compound to bind covalently and inhibit the receptor in a number of in vitro experiments, and provide evidence for its point of attachment to the receptor.

## Methods and materials

### Chemicals and reagent

The radioligand [^3^H] ZM241385 with a specific activity of 50 Ci × mmol^−1^ was purchased from ARC Inc. (St. Louis, MO). Unlabelled ZM241385 was a gift from Dr. S.M. Poucher (Astra Zeneca, Macclesfield, UK). 5′-N-ethylcarboxamidoadenosine (NECA) was purchased from Sigma-Aldrich (Steinheim, Germany). LUF6632 was synthesized in our lab, as published previously [19]. Adenosine deaminase (ADA) was purchased from Boehringer Mannheim (Mannheim, Germany). Bicinchoninic acid (BCA) and BCA protein assay reagent were obtained from Pierce Chemical Company (Rockford, IL, USA). HEK293 cells stably expressing the hA_2A_ adenosine receptor (HEK293 hA_2A_AR) were kindly provided by Dr. J Wang (Biogen/IDEC, Cambridge, MA, USA). All other chemicals were of analytical grade and obtained from standard commercial sources.

### Site-directed mutagenesis

Site-directed receptor mutant hA_2A_AR-K153A^ECL2^ was constructed by the same procedure reported previously [20]. The wild type pcDNA3.1-A_2A_R plasmid DNA with N-terminal HA and FLAG tags and C-terminal His tag was used as a template for polymerase chain reaction (PCR) mutagenesis. Mutant primers for directional PCR product cloning were designed using the online Quickchange primer design program (Agilent Technologies, Santa Clara, CA), and primers were obtained from Eurogentec (Maastricht, The Netherlands). All DNA sequences were verified by Sanger sequencing at LGTC (Leiden, The Netherlands).

### Cell culture, transfection, and membrane preparation

We followed the procedures reported previously [20, 21]. Briefly, human embryonic kidney (HEK) 293 cells were grown as monolayers in Dulbecco’s modified Eagle’s medium supplemented with stable glutamine, 10% newborn calf serum, 50 μg/mL streptomycin, and 50 IU/mL penicillin at 37 °C and 7% CO_2_ atmosphere. The cells were transfected with mutant plasmid DNA using the calcium phosphate precipitation method, followed by a 48-h incubation. And HEK293 hA_2A_AR wild type (hA_2A_AR-WT) cells were grown as monolayers on 15 cm ø culture plates to 80–90% confluency in the same medium as the other HEK293 cells but with the addition of G-418 (500 mg/ml). For both cells were detached from the plates by scraping them into PBS and centrifuged to remove PBS buffer. The pellets were resuspended in ice-cold Tris-HCl buffer (50 mM, pH 7.4) and then homogenized. The cell membrane suspensions were centrifuged at 100,000×*g* at 4 °C for 20 min, after which the procedure was repeated one more time. After this, Tris-HCl buffer was used to resuspend the pellet, and adenosine deaminase was added to break down endogenous adenosine. Membranes were stored in 250 μL aliquots at −80 °C until further use. Membrane protein concentrations were measured using the BCA method [22].

### Radioligand displacement assay

Radioligand displacement experiments were performed as follows. Membrane aliquots containing 10 μg of protein were incubated in a total volume of 100 μL of assay buffer to adjust the assay window to approximately 3000 dpm. Nonspecific binding was determined in the presence of 100 μM NECA and represented less than 10% of the total binding. Then, to each tube were added 25 μL cell membrane (10 μg of protein), 25 μL of 2.7 nM radioligand [^3^H] ZM241383, 25 μL of assay buffer [25 mM Tris-HCl, pH 7.4 at 25 °C , supplemented with 5 mM MgCl_2_ and 0.1% (*w*/*v*) 3-[(3-cholamidopropyl) dimethylammonio]-1-propanesulfonate (CHAPS)], and 25 μL of the indicated compounds in increasing concentrations in the same assay buffer. The mixture was incubated at 25 °C for 60 min to reach equilibrium. Incubations were terminated by rapid vacuum filtration to separate the bound and free radioligand through 96-well GF/B filter plates using a Perkin Elmer Filtermate-harvester (PerkinElmer, Groningen, Netherlands). Filters were subsequently washed three times with 2 mL of ice-cold buffer (25 mM Tris-HCl, pH 7.4, supplemented with 5 mM MgCl_2_). The filter-bound radioactivity was determined by scintillation spectrometry using a P-E 1450 Microbeta Wallac Trilux scintillation counter (PerkinElmer).

### Radioligand competition association assay

The binding kinetics assay to determine the unlabeled ligands was performed as described previously [21]. Briefly, the association of the radioligand was followed over time in the absence or presence of a concentration corresponding to the IC_50_ value of unlabeled ZM241385, LUF6632, and LUF7445. In practice, to the mixture of equal volumes of radioligand, unlabeled compound and assay buffer was added a 25 μL membrane aliquot containing 10 μg of protein at each time point from 0.5 min to 240 min at 25 °C. Incubation was terminated as described above (Radioligand displacement assay).

### Irreversible binding of LUF7445 to both hA_2A_AR-WT and hA_2A_AR-K153A^ECL2^ cell membranes

Both hA_2A_AR-WT and hA_2A_AR-K153A^ECL2^ cell membrane aliquots were treated the same way as described in the “Radioligand displacement assay” section to determine their assay window. Then, 100 μL assay buffer containing either 1% DMSO (as blank control for total binding and nonspecific binding) or 1 μM ligands (ZM241385 or LUF7445, 400 μM stock in assay buffer) was added to 2-mL Eppendorf tubes containing 100 μL cell membrane suspension and 200 μL assay buffer and incubated for 1 h at 25 °C. Subsequently, the mixture was centrifuged at 16,100×*g* at 4 °C for 5 min to remove the buffer with the “free” ligands. The membrane pellet was resuspended in 1 mL assay buffer and spun for 5 min at 16,100×*g* at 4 °C. After three washing cycles, the cell pellets were resuspended in 300 μL assay buffer to determine their radioligand binding activity. Afterwards, all the samples were transferred to test tubes on ice and 100 μL (2.7 nM) radioligand [^3^H] ZM241383 was added, followed by a 0.5-h incubation at 25 °C. The incubation was terminated by vacuum filtration through a GF/B filter using a Brandel harvester to separate bound and free radioligand. The filters were washed three times with ice-cold wash buffer (25 mM Tris-HCl, pH 7.4 supplemented with 5 mM MgCl_2_). After harvesting, 3.5 mL of scintillation liquid was added and the filter-bound radioactivity was determined in a Tri-Carb 2900TR liquid scintillation analyzer (PerkinElmer). Results are expressed as percentage normalized to the maximum specific binding in the control group (100%).

### Cyclic AMP functional assay

The LANCE ultra-cAMP 384 kit (PerkinElmer, Groningen, Netherlands) was used and all assay components were prepared according to the instructions of the manufacturer. Briefly, cAMP was generated in the stimulation buffer (*N*-2-hydroxyethylpiperazine-*N*-ethane sulfonic acid (HEPES), 5 mM; 0.1% (*w*/*v*) BSA; cilostamide, 50 μM; rolipram, 50 μM; adenosine deaminase (ADA), 0.8 IU mL^−1^). HEK293 hA_2A_AR cells were grown as monolayers to 80–90% confluency and harvested by centrifugation for 5 min at 200×*g*. Then, 5000 cells per well were seeded in a 384 well plate, followed by a 1-h co-incubation with a mixture of 10 nM NECA (prepared in the stimulation buffer) and the antagonists (LUF7445 or ZM241385) at a concentration ranging from 1 μM to 1 pM. Then, the incubation was terminated by adding cAMP Tracer solution and anti-cAMP solution. Measurements of the generated fluorescence intensity were done on an EnVision Multilabel Reader (PerkinElmer, Groningen, Netherlands).

### Irreversible binding of LUF7445 to HEK293 hA_2A_AR cells assessed in cyclic AMP functional assay

All the assay components were prepared as described in the cAMP functional assay above. HEK293 hA_2A_AR cells were grown as monolayers to 80–90% confluency and harvested by 200×*g* centrifugation for 5 min. Then, cells were pretreated with ligands at the concentration of their IC_80_ values (determined in the cAMP functional assay above), or with stimulation buffer (pH 7.4) for 1 h. Then, the pretreated cells were centrifuged for 5 min at 300×*g* to remove the supernatant at 4 °C, after which the cell pellet was washed three times with 3 × 1 mL stimulation buffer, separated by renewed incubation for 10 min at 25 °C. These washed cells were seeded in a 384 well plate (5000 cells/well) as described in the cAMP functional assay above. Briefly, 10 nM NECA (prepared in the stimulation buffer) was co-incubated to stimulate cAMP production, followed by the termination by cAMP Tracer solution and anti-cAMP solution. Measurements of the generated fluorescence intensity were done on an EnVision Multilabel Reader (PerkinElmer, Groningen, Netherlands).

### Computer modeling

All calculations were performed using the Schrodinger Suite [23]. The high-resolution crystal structure of the adenosine A_2A_ receptor co-crystalized with a ZM241385 was used for the docking studies (PDB:4EIY) [14]. The crystal structure was prepared using the preparation wizard; protonation states were assigned using PROPKA [24]. After the protein preparation, we used the CovDock [25] module to perform covalent docking on residue LYS153EL2. Figures were rendered using PyMol [26].

### Data analysis

All the experimental data were analyzed with GraphPad Prism 6.0 software (GraphPad Software Inc., San Diego, CA). The radioligand displacement curves were fitted to a one-site binding model. Association data for the radioligand were fitted using one-phase exponential association. Values for *k*_on_ were obtained by converting *k*_obs_ values using the following equation: *k*_on_ = (*k*_obs_ − *k*_off_ )/[radioligand], where *k*_off_ values were cited from Guo et al. [21] Association and dissociation rates for unlabelled ligands were calculated by fitting the data in the competition association model using “kinetics of competitive binding” [21, 27].

$$ {K}_A={k}_1\left[L\right]+{k}_2 $$

$$ {K}_B={k}_3\left[L\right]+{k}_4 $$

$$ S=Sqrt\left[{\left({K}_A-{K}_B\right)}^2+4\times {k}_1\times {k}_3\times L\times I\right] $$

$$ {K}_F=0.5\left({K}_A+{K}_B+S\right) $$

$$ {K}_s=0.5\left({K}_A+{K}_B-S\right) $$

$$ Q={B}_{\max}\times {k}_1\times L\times {\left({K}_F-{K}_S\right)}^{-1} $$

$$ Y=Q\times \left[{k}_4\times \left({K}_F-{K}_S\right)\times {K_F}^{-1}\times {K_S}^{-1}+\left({k}_4{\textstyle -}{K}_F\right)\times {K_F}^{-1}\times {e}^{-{K}_F\times X}-\left({k}_4-{K}_S\right)\times {K_S}^{-1}\times {e}^{-{K}_S\times X}\right] $$

where *X* is the time (min), *Y* is the specific [^3^H]-ZM241385 binding (DPM), *k*_1_ and *k*_2_ are the *k*_on_ (nM^−1^ min^−1^) and *k*_off_ (min^−1^) of [^3^H]-ZM241385 and were obtained from Guo et al. [21], *L* is the concentration of [^3^H]-ZM241385 used (nM), *B*_max_ the total binding (DPM), and *I* the concentration of unlabeled ligand (nM). Fixing these parameters allows the following parameters to be calculated: *k*_3_, which is the *k*_on_ value (nM^−1^ min^−1^) of the unlabeled ligand and *k*_4_, which is the *k*_off_ value (min^−1^) of the unlabeled ligand. The residence time (RT) was calculated using RT = 1 / k_off_ [28]. Functional concentration-effect curves were fitted to a three-parameter concentration response model. Values are expressed as mean ± S.E.M. of three independent experiments performed in duplicate. Statistical analyses were performed using Student’s unpaired *t* test (***P < 0.001, **P < 0.01, *P < 0.05).

## Results

### Design and synthesis of LUF7445

**Fig. 1:**
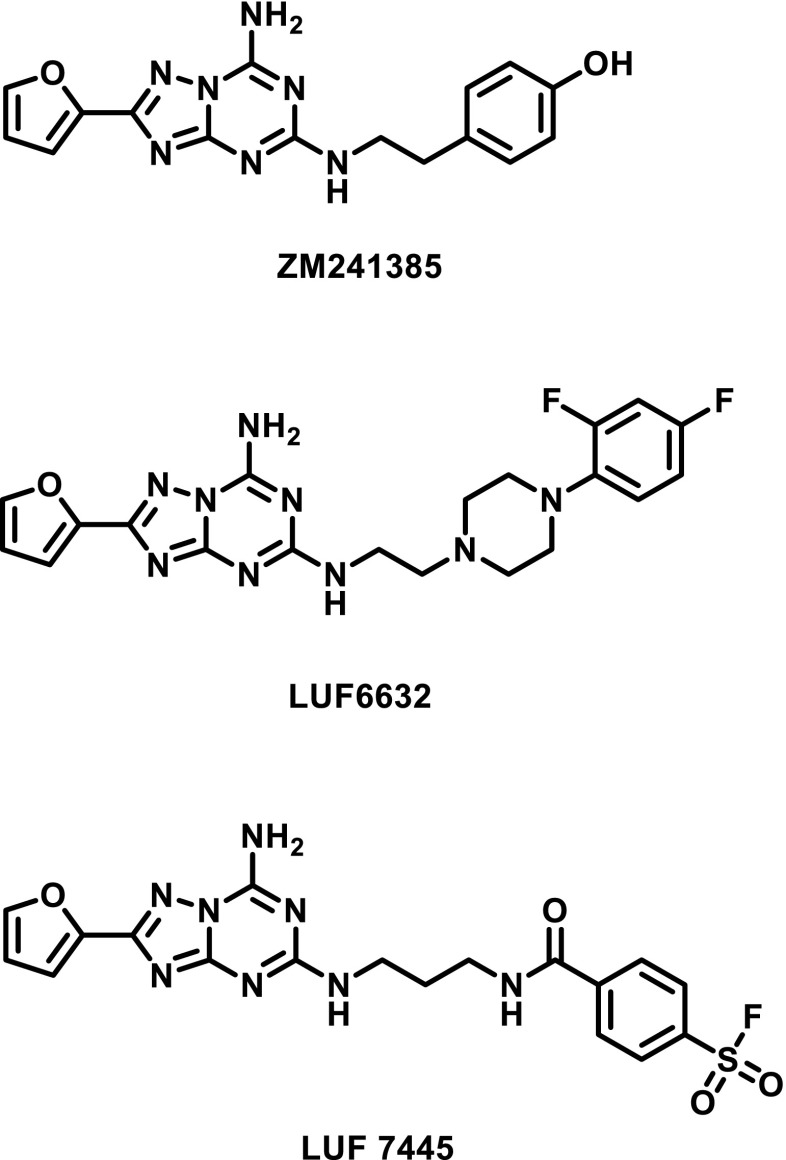
Chemical structures of the three hA_2A_ receptor antagonists examined in this study

Over the years, our research group has explored series of triazolotriazine derivatives based on the reference adenosine A_2A_ antagonist ZM241385, 4-(2-(7-amino-2- (furan-2-yl)- [1, 2, 4]triazolo[1,5-*a*] [1, 3]triazin-5-ylamino)ethyl)phenol (Fig. 1), to investigate their structure-activity and structure-kinetics relationships (SAR and SKR) [22, 29]. We identified LUF6632 (Fig. 1) as a long residence time (RT) compound compared to other derivatives. This compound prompted us to bring the concept of prolonged receptor occupancy further by aiming for a covalently binding derivative of ZM241385. Hence, LUF 7445 (Fig. 1), 4-((3-((7-amino-2-(furan-2-yl)- [1, 2, 4]triazolo[1,5-*a*] [1, 3]triazin-5-yl)amino)propyl)carbamoyl)benzene sulfonyl fluoride was synthesized in three steps from sulfone compound 2-(furan-2-yl)-5-(methylsulfonyl)- [1, 2, 4] triazolo[1,5-*a*][1, 3]triazin-7-amine as starting reagent. The reaction conditions and other reagents used are described in synthetic Scheme 1 of the SI.

### Determination of the affinity (*K*_i_) of LUF7445, LUF6632, and ZM241385 for the A_2A_ receptor

**Fig. 2:**
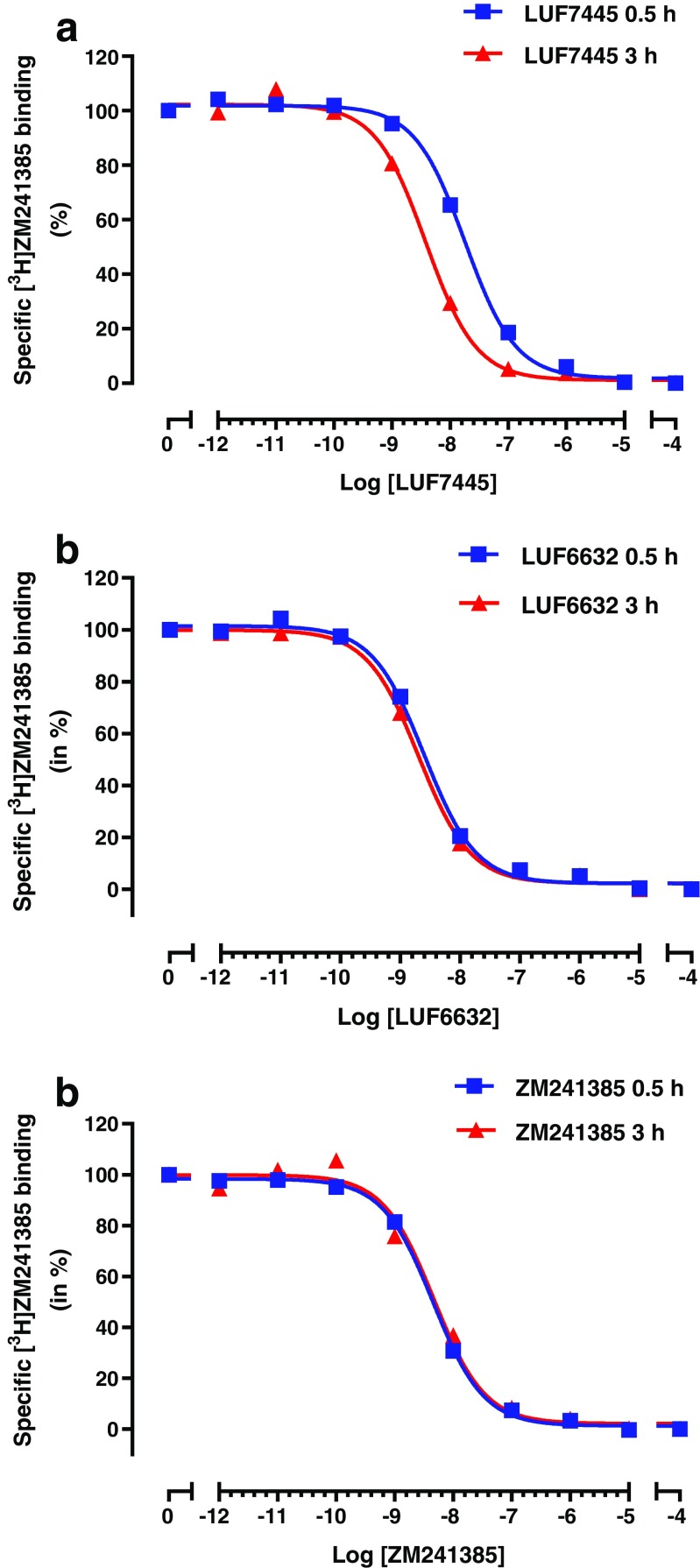
Displacement of specific [^3^H] ZM241385 binding from the adenosine hA_2A_AR receptor at 25 °C by LUF7445 (**a**), LUF6632(**b**), and ZM241385(**c**) during an incubation of 0.5 h (*blue curve*) and 3 h (*red curve*), respectively. Representative graphs are from one experiment performed in duplicate

To determine the affinity (*K*_i_) for the A_2A_ receptor LUF7445, LUF6632 and ZM241385 were tested in a [^3^H] ZM241385 displacement experiment (*n* = 3). All these compounds concentration-dependently inhibited specific [^3^H] ZM241385 binding from human A_2A_ receptors overexpressed in HEK293 cell membranes (Fig. 2). LUF6632, ZM241385, and LUF7445 showed similar affinities in the subnanomolar range (Table 1). It should be mentioned that the putative covalent nature of the interaction between receptor and LUF7445 precludes the determination of equilibrium binding parameters. Therefore, we expressed LUF7445’s affinity for the A_2A_ receptor as “apparent *K*_i_” (*K*_i_^*^).

**Table 1:** (Apparent) affinities of LUF7445, LUF6632, and ZM241385 for the A_2A_ adenosine receptor

Compound	p*K*_i_^a^ (0.5 h)	p*K*_i_^b^ (3 h)
LUF7445^c^	8.27 ± 0.042	8.99 ± 0.008***
LUF6632	9.17 ± 0.007	9.26 ± 0.004*
ZM241385	8.89 ± 0.019	8.91 ± 0.006

Data are expressed as means ± SEM of three separate experiments each performed in duplicate. *P < 0.05, ***P < 0.001 compared with the p*K*_i_ values in displacement experiments during 0.5 h incubation time; Student’s *t* test

^a^Affinity determined from displacement of specific [^3^H]ZM241385 binding from the hA_2A_AR at 25 °C during 0.5 h incubation

^b^Affinity determined from displacement of specific [^3^H]ZM241385 binding from the hA_2A_AR at 25 °C during 3 h incubation

^c^For LUF7445, the covalent antagonist, p*K*i values can only be apparent, as true equilibrium cannot be reached

### Time-dependent characterization of affinity for LUF7445, LUF6632 and ZM241385

We then tested the time dependency of the affinity of the three compounds. To that end, a [^3^H] ZM241385 displacement experiment was performed with an incubation time of both 0.5 and 3 h. As detailed in Table 1, the affinity of LUF6632 slightly and of LUF7445 strongly increased with longer incubation time while ZM241385’s affinity did not change. Representative graphs for this effect are in Fig. 2, in which the curve representing a concentration-dependent inhibition of specific [^3^H] ZM241385 binding was shifted to the left with time for LUF7445 (Fig. 2a), with little (LUF6632, Fig. 2b) or no difference (ZM241385, Fig. 2c) for the other two compounds. Notably, compared to the long residence compound LUF6632, LUF7445 showed a more pronounced influence with prolonged incubation time, suggesting an increasing level of covalent binding over time. The combined data yielded an approx. fivefold shift in apparent *K*_i_ value for LUF7445. The affinities of the compound for the other adenosine receptor subtypes are reported in Table S1 of the SI, showing that LUF7445 is very selective towards A_2A_ receptors.

### Kinetic characterization of LUF6632, LUF7445, and ZM241385 in a competition association binding assay

**Fig. 3:**
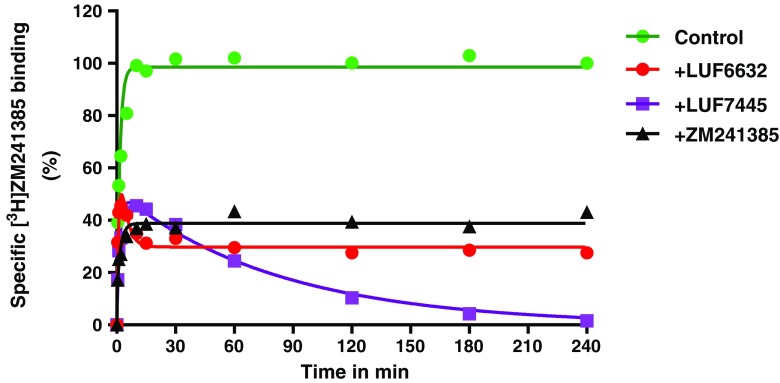
Competition association binding assay with [^3^H] ZM241385 in the absence or presence of indicated compounds at 25 °C. Representative graphs are from one experiment performed in duplicate (see Table 2 for kinetic parameters)

The “apparent *K*_i_ shift” of LUF7445 drove us to investigate the irreversible characteristics of LUF7445 binding by performing kinetic assays to determine its dissociation rate from the A_2A_ adenosine receptor. In our previous research, the *k*_on_ (*k*_1_ = 0.24 ± 0.05 × 10^8^ M^−1^ min^−1^) and *k*_off_ (*k*_2_ = 0.48 ± 0.03 min^−1^) values of [^3^H]-ZM241385 at 25 °C have been determined by a traditional association and dissociation assay [21]. Here, we derived the kinetic parameters, i.e., the *k*_on_ (*k*_3_) and *k*_off_ (*k*_4_) values, for the three unlabeled ligands from a competition association assay (Fig. 3). Both LUF6632 (*k*_on_ = 1.53 ± 0.083 nM^−1^ min^−1^) and ZM241385 (*k*_on_ = 1.72 ± 0.36 nM^−1^ min^−1^) showed a similar association rate, which was significantly faster than for LUF7445 (*k*_on_ = 0.0059 ± 0.00049 nM^−1^ min^−1^). As detailed in Table 2, LUF6632 displayed a dissociation rate constant of 0.15 ± 0.021 min^−1^ which equals to a receptor RT of 6.80 ± 0.97 min, being sevenfold longer than ZM241385’s RT which was 0.96 ± 0.12 min at 25 °C. Figure 3 shows that LUF7445’s behavior was very different, causing an initial “overshoot” of the competition association curve which over time progressed to negligible radioligand binding at 240 min. Analyzing this curve with the (equilibrium), Motulsky and Mahan model [27] led to a negligible dissociation rate (*k*_off_ = 1.37 ± 0.68 × 10^−11^ min^−1^) and an almost infinite RT for LUF7445 (values between brackets in Table 2). These data provided further evidence for a putative irreversible binding mode between LUF7445 and the hA_2A_ receptor. This data is qualitatively summarized in a simplified scheme in the SI (Scheme 2).

**Table 2:** The (apparent) association and dissociation rate constants of LUF7445, LUF6632, and ZM241385 determined in competition association assays with [^3^H]-ZM241385 binding to HEK293-hA_2A_AR membranes

Compound	*k*_on_ (nM^−1^ min^−1^)^a^	*k*_off_ (min^−1^)^a^	RT (min)
LUF7445^b^	(0.0059 ± 0.00049)	(1.37 ± 0.68 × 10^−11^)	(2.86 ± 0.87 × 10^11^)
LUF6632	1.53 ± 0.083	0.15 ± 0.021	6.80 ± 0.97
ZM241385	1.72 ± 0.36	1.04 ± 0.13	0.96 ± 0.12

^a^Association (*k*_on_) and dissociation (*k*_off_) rate constants were determined by competition association assay at 25 °C; all these values were determined by analyzing the data in the mathematical model described by Motulsky and Mahan [27]

^b^For LUF7445, no equilibrium is reached between receptors and ligand; hence, the Motulsky/Mahan mathematical model [27] for the competition association assay is not valid. The values obtained are therefore considered to provide qualitative insight only, and are in brackets

### Binding mode of LUF7445 in the hA_2A_AR binding pocket

**Fig. 4:**
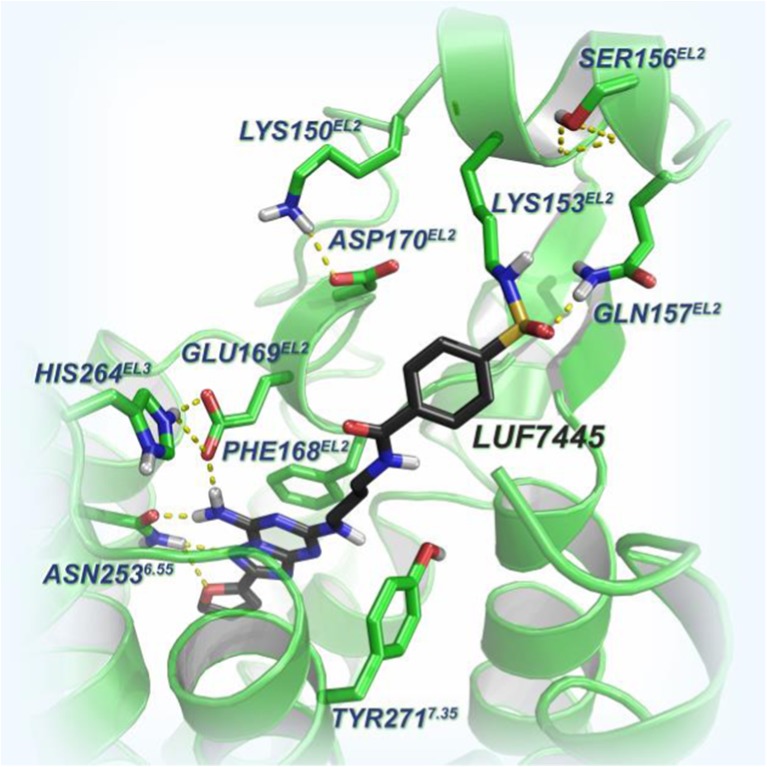
Binding model of LUF7445 in the hA_2A_ adenosine receptor-binding pocket based on the hA_2A_ adenosine receptor crystal structure (PDB code: 4EIY). The *black carbon sticks* represent the structure of LUF7445. The important residues and H–bonds for ligand recognition are indicated by *yellow dashed lines*

Although the radioligand binding results above characterized LUF7445 as an irreversibly binding ZM241385 derivative, it remained to be tested what the target residue of the reactive warhead is. We therefore constructed a binding model based on the reported adenosine A_2A_ X-ray crystal structure (PDB code: 4EIY) and chemical structure of LUF7445. From the docking result, the ZM241385 core structure, shown as black carbon sticks in Fig. 4, is in the same position as ZM241385 in the A_2A_ crystal structure, participating in H–bond formation with residues such as His264, Glu169, Phe168, and Asn253. Due to the flexibility of the three carbon linker, a lysine residue in close proximity of the ligand, K153^ECL2^, could interact with the 4-fluorosulfonylbenzoic warhead in LUF7445 to form a covalent sulfonyl amide.

### Lysine K153^ECL2^ residue is the possible anchor point for covalent bond formation

**Fig. 5:**
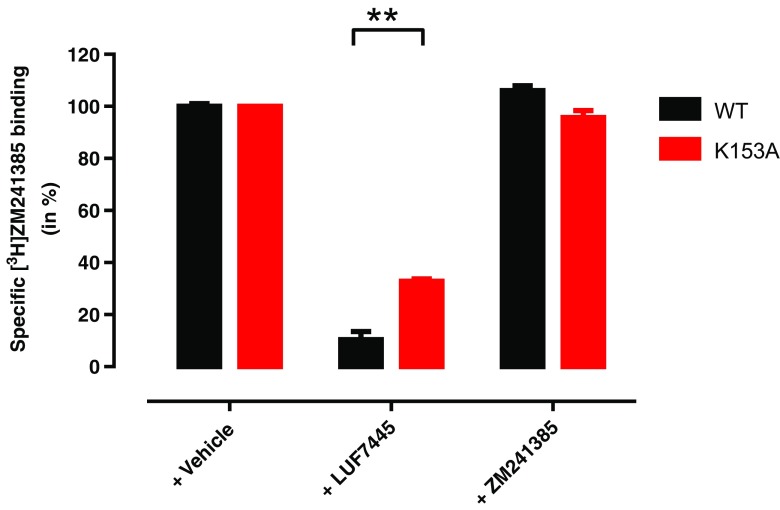
Involvement of Lys153 in the binding of LUF7445. HEK293 cell membranes overexpressing wild-type or K153A mutant hA_2A_ AR were pretreated with buffer (vehicle) or 1 μM of LUF7445 or ZM241385 for 1 h followed by 3 wash cycles. The membranes were then subjected to a standard [^3^H] ZM241385 radioligand binding assay to measure remaining specific [^3^H] ZM241385 binding. Results were obtained from three independent experiments performed in duplicate. Data are normalized to 100% of the vehicle group response. *Error bars* indicate SEM values.**Significant difference between groups (*P* < 0.01); Student’s *t* test

To investigate the structural nature of the interaction between the ligands and receptor, we therefore mutated the potential target lysine residue to alanine (A_2A_AR-K153A^ECL2^ receptor) to compare with the wild-type receptor and perform a “wash-out” experiment. Following preincubation with either LUF7445 or ZM241385, cell membranes overexpressing mutant A_2A_AR-K153A^ECL2^ or wild-type A_2A_AR were washed three times to remove the noncovalently bound ligands. After this repeated washing, cell membranes were incubated with the radioligand [^3^H] ZM241385 to assess the remaining radioligand binding. In the absence of antagonist (labeled “+ vehicle” in Fig. 5) both the mutant A_2A_AR-K153A^ECL2^ and the wild-type A_2A_AR receptor containing membranes had a similar recovery of radioligand binding, which we normalized to 100% recovery. LUF7445 caused a significant decrease of radioligand binding on the A_2A_AR WT cell membranes with only 10.4 ± 3.0% recovery of specific binding despite the extensive washing, while more radioligand binding was “saved” at the cell membranes overexpressing A_2A_AR-K153A^ECL2^ (32.8 ± 0.9% remaining). As a control, both cell membrane preparations preincubated with ZM241385 showed that ZM241385 was rapidly washed off the membranes, as a full recovery of radioligand binding was observed.

### Functional characterization of LUF7445 and ZM241385 in cAMP assay

**Fig. 6:**
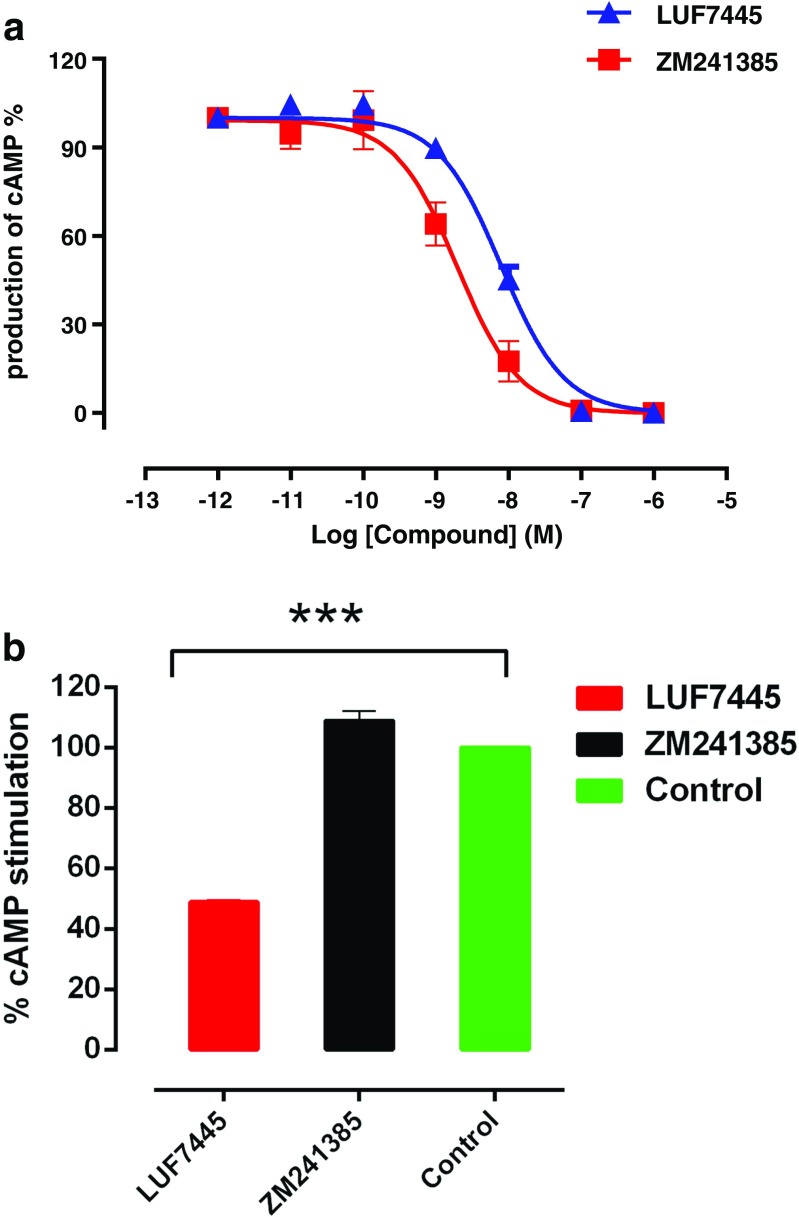
Functional characterization of LUF7445 and ZM241385 on hA_2A_ AR expressed in HEK293 cells. **a** Concentration-inhibition curves for LUF7445 and ZM241385 in a cAMP assay in the presence of 10 nM NECA (100%). Results were obtained from three independent experiments performed in triplicate. **b** Recovery of cAMP production. Cells were pretreated with a concentration corresponding to the IC_80_ value (retrieved from Fig. 6a) of the indicated compound, or with buffer (control) for 1 h. Then, 3 wash cycles were applied, followed by adding 10 nM NECA to stimulate cAMP production. Data are expressed as means ± SEM of three separate experiments each performed in duplicate. ***Significant difference between groups (*P* < 0.001); Student’s *t* test

Functional characterization of these compounds in a cAMP assay on the HEK cells expressing the hA_2A_AR showed their antagonist behavior. The cAMP production was stimulated by the addition of the reference agonist NECA (10 nM). Both LUF7445 and ZM241385 caused a concentration-dependent inhibition of NECA’s effect (100% in the absence of antagonist, see Fig. 6a). The potency of LUF7445 (pIC_50_ = 8.10 ± 0.044) was somewhat lower than of ZM241385( pIC_50_ = 8.71 ± 0.13). Again, it should be mentioned that LUF7445 precludes a true equilibrium affinity determination. From Fig. 6a, we determined the IC_80_ values of the two compounds, which concentrations were then used to pretreat the HEK-A_2A_AR cells, followed by three wash steps. Thereafter, we stimulated the cAMP production in these cells with 10 nM NECA, resulting in a sustained inhibition of cAMP production in the presence of LUF7445 (48 ± 1%), while ZM241385 showed no difference in restoration of cAMP production compared to the control cells in the absence of any indicated compound (Fig. 6b). Apparently, the cAMP production induced by NECA in the presence of LUF7445 was inhibited under conditions where a reference antagonist did not, further validating LUF7445 as a covalent antagonist forming an irreversible bond with the hA_2A_AR.

## Discussion

Covalent ligands for GPCRs are emerging as a useful tool for receptor structure elucidation and the chartering of the ligand-receptor binding pocket. As an example, the 3D architecture of the beta_2_-adrenergic receptor in an active conformation has been recently determined in the presence of a covalently binding derivative of noradrenaline [4, 30]. In the current study, we designed and synthesized a covalent antagonist (LUF7445) to investigate ligand-receptor interaction in the binding pocket of the hA_2A_AR, and compared its behavior to the reference antagonist ZM241,385 and the long residence time ZM-derivative LUF6632 [19]. All three compounds showed a high affinity for the hA_2A_AR.

The rational ligand design came from the reported crystal structures of the hA_2A_AR bound to ZM241385, providing a clear blueprint of ligand binding interactions [13, 14, 31]. A deep, planar, and narrow cavity holds the aromatic core and furan ring of ZM241385, while the phenylethylamine substituent is directed to the extracellular region (EL2 and EL3). The architecture of the ligand binding pocket offered us a good starting point for the structural modification of ZM241385. Therefore, instead of the 4-hydroxyphenylethylamine side chain in ZM241,385, the electrophilic fluorosulfonyl group, chosen to permit a possible nitrogen-to-sulfur bond between the ligand and a nearby free amino group in the receptor, was introduced and incorporated in a linker to yield LUF7445.

A first hint of the covalent nature of LUF7445 was found in incubation time-dependent radioligand displacement assays. A longer incubation time rendered LUF7445 more potent in displacing the radioligand from the receptor, while this was not or hardly the case for ZM241384 and LUF6632, respectively. LUF6632 had previously been identified as an antagonist with a long residence time (>300 min) at the receptor, when assessed at 4 °C [19]. The current set of experiments was performed at 25 °C, making LUF6632 dissociate faster (RT = 6.8 min, Table 2) from the receptor with no substantial p*K*_i_ shift in affinity at the two incubation times. Similar experiments on other GPCRs, such as CB_1_ cannabinoid receptor [32, 33] and histamine H_2_ receptor [34], demonstrated that the covalent interaction between the ligand and the receptor resulted in a time-dependent affinity change.

However, it is far from conclusive to identify a presumed covalent ligand from an affinity shift alone, as pseudo-irreversible interactions can also occur caused by slow dissociation rates. From a kinetic perspective, a covalent ligand refers to a ligand that stays at the receptor for an infinite time period. If the incubation time is long enough, all receptors will be occupied by the covalent compound, rendering competitive radioligand binding impossible. In accordance with this, a continuing decrease of specific radioligand binding was observed in the kinetic experiments over a 4-h incubation at 25 °C (Fig. 3). The inadequacy of the Motulsky-Mahan equations [27] to fit this data is further evidence for the nonequilibrium features of the binding of LUF7445 to the receptor. Furthermore, extensive washing did not free the receptors from LUF7445, as demonstrated by the lack of [^3^H]ZM241385 binding (Fig. 5, WT receptor), compared to a full recovery of membranes pretreated with ZM241385. This confirms the washing steps did remove the reversible ligand from the receptors, and in return validates the irreversible binding of LUF7445 to the hA_2A_AR. Similar findings were obtained on the adenosine A_1_ receptor and histamine H_4_ receptor where preincubation of a covalently binding ligand concentration-dependently decreased radioligand binding after extensive washing of the cell membrane preparation [7, 35].

Based on the ZM241385 binding mode of the hA_2A_AR, we hypothesized that LUF7445 covalently interacts with a lysine residue, K153^ECL2^, resulting in a sulfonamide bond formation (Fig. 4). Hence, the K153A^ECL2^ mutant construct, potentially preventing the covalent bond from being formed, was made to perform a similar wash-out experiment as described above. Since ZM241385 showed a similar affinity for both the K153A^ECL2^ mutant (p*K*i = 7.83 ± 0.04) and WT receptors (p*K*i = 7.91 ± 0.05) [20], we assumed that the difference in radioligand binding recovery was not due to a point mutation within a receptor binding site, which has the potential of altering ligand binding properties. Moreover, in the absence of either LUF7445 or ZM241385, the apparently same binding capacity (data not shown) proved that the washing steps had little influence on the integrity of both WT hA_2A_AR and mutant A2AAR-K153A^ECL2^. The mutation led to a threefold increase of binding recovery indicating a substantially decreased level of covalent binding to the cell membranes, resulting from a decreased possibility to form a covalent bond between the warhead and a target residue. The mutation did not lead to a full recovery of radioligand binding, however, suggesting that other unidentified residues may play a similar role. Likewise, Nijmeijer et al. identified a cysteine amino acid to be the linking residue for the covalent probe at the histamine H_4_ receptor mentioned above [35], and it may be that the very reactive fluorosulfonyl warhead in LUF7445 also targets other residues such as cysteines [36].

A covalent antagonist will decrease the maximal agonist-induced effect by a permanent occupancy of the available receptors, which was indeed demonstrated in the functional assay. The concentration-effect curves obtained in the cAMP functional assay showed an antagonistic behavior of LUF7445 (Fig. 6a). A much lower stimulation by AR receptor agonist NECA was observed as the number of available receptors was most likely reduced by the irreversible binding of LUF7445 (Fig. 6b). A similar experiment at the adenosine A_1_ receptor showed that the irreversible binding by FSCPX decreased the maximal effect in the agonist dose-response curves [37]. All these results contributed to our hypothesis that LUF7445 is an insurmountable antagonist for the hA_2A_AR indeed, and that the fluorosulfonyl group present in LUF7445 reacts with K153^ECL2^ via a covalent modification.

Besides in clinical trials for Parkinson’s disease, A_2A_ antagonists have risen to prominence as a future add-on to cancer combination therapy. Under chronic hypoxic conditions within the tumor microenvironment, increased accumulation of extracellular adenosine around the tumor tissue activates A_2A_ARs in the vicinity, which promotes peripheral tolerance by inducing T-cell anergy and the generation of adaptive regulatory T cells [38]. In contrast, blockade of A_2A_ARs plays a vital role in retardation of tumor growth, relieving immune cells from their repressed conditions, reducing the metastasis of tumors [39], and thus boosting antitumor immunity. As a consequence potent, A_2A_ receptor antagonists are now being considered as potential therapeutics in diminishing the rate of cancer development [40, 41]. The starting point of our design strategy, ZM241385, has been reported to significantly inhibit melanoma growth and reinforce the antineoplastic immune response, when combined with anti-CTLA4mAb [42]. However, in vivo tumor rejection during treatment with ZM241385 failed to take place most likely because of ZM241385’s short half-life [11]. In addition, we speculate that the relatively short receptor residence time of ZM241385 at physiological temperature is another aspect that allows the massive amounts of adenosine produced in the tumor environment to continue to activate the A_2A_AR. Thus, a covalently binding antagonist such as LUF7445 may be a better proposition under these conditions.

## Conclusion

The structure-based design of LUF7445, an antagonist for the human A_2A_AR, is reported in this study. In a number of in vitro assays, we obtained accumulating evidence for the covalent nature of the ligand’s interaction with the receptor. More specifically, LUF7445 appeared to bind covalently to a lysine residue in the extracellular domain of the receptor (K153^ECL2^). Its antagonistic nature was confirmed in a functional assay, as it blocked hA_2A_AR-mediated cAMP accumulation by agonist NECA. The results contribute to a better understanding of long-lasting effects caused by ligands covalently reacting/interacting with GPCRs. In itself, LUF7445 may be a probe to explore the added value of covalent antagonists for the adenosine A_2A_ receptor in certain disease states such as cancer immunology, in which high adenosine levels are causative. In the end, rational design of covalent probes may have further value in new technologies such as activity-based protein profiling with the perspective of imaging or structural probing of GPCRs.

## Electronic supplementary material

ESM 1 (DOCX 122 kb)

## References

[1] Overington JP, Al-Lazikani B, Hopkins AL (2006). Opinion—how many drug targets are there?. Nat Rev Drug Discov.

[2] Katritch V, Cherezov V, Stevens RC (2013). Structure-function of the G protein-coupled receptor superfamily. Annu Rev Pharmacol Toxicol.

[3] Rosenbaum DM, Rasmussen SGF, Kobilka BK (2009). The structure and function of G-protein-coupled receptors. Nature.

[4] Lagerstrom MC, Schioth HB (2008). Structural diversity of G protein-coupled receptors and significance for drug discovery. Nat Rev Drug Discov.

[5] Jacobson KA, Barone S, Kammula U, Stiles GL (1989). Electrophilic derivatives of purines as irreversible inhibitors of A_1_-adenosine receptors. J Med Chem.

[6] Srinivas M, Shryock JC, Scammells PJ, Ruble J, Baker SP, Belardinelli L (1996). A novel irreversible antagonist of the A_1_-adenosine receptor. Mol Pharmacol.

[7] van Muijlwijk-Koezen JE, Timmerman H, van der Sluis RP, van de Stolpe AC, Menge WMPB, Beukers MW, van der Graaf PH, de Groote M, IJzerman AP (2001). Synthesis and use of FSCPX, an irreversible adenosine A_1_ antagonist, as a ‘receptor knock-down’ tool. Bioorg Med Chem Lett.

[8] Shryock JC, Snowdy S, Baraldi PG, Cacciari B, Spalluto G, Monopoli A, Ongini E, Baker SP, Belardinelli L (1998). A_2A_-adenosine receptor reserve for coronary vasodilation. Circulation.

[9] Ruiz MD, Lim YH, Zheng JY (2014). Adenosine A_2A_ receptor as a drug discovery target. J Med Chem.

[10] Schwarzschild MA, Agnati L, Fuxe K, Chen JF, Morelli M (2006). Targeting adenosine A_2A_ receptors in Parkinson's disease. Trends Neurosci.

[11] Ohta A, Gorelik E, Prasad SJ, Ronchese F, Lukashev D, Wong MKK, Huang XJ, Caldwell S, Liu KB, Smith P, Chen JF, Jackson EK, Apasov S, Abrams S, Sitkovsky M (2006). A_2A_ adenosine receptor protects tumors from antitumor T cells. P Natl Acad Sci USA.

[12] Xu F, Wu HX, Katritch V, Han GW, Jacobson KA, Gao ZG, Cherezov V, Stevens RC (2011). Structure of an agonist-bound human A_2A_ adenosine receptor. Science.

[13] Jaakola VP, Griffith MT, Hanson MA, Cherezov V, Chien EYT, Lane JR, IJzerman AP, Stevens RC (2008). The 2.6 angstrom crystal structure of a human A_2A_ adenosine receptor bound to an antagonist. Science.

[14] Liu W, Chun E, Thompson AA, Chubukov P, Xu F, Katritch V, Han GW, Roth CB, Heitman LH, IJzerman AP, Cherezov V, Stevens RC (2012). Structural basis for allosteric regulation of GPCRs by sodium ions. Science.

[15] Barrington WW, Jacobson KA, Hutchison AJ, Williams M, Stiles GL (1989). Identification of the A_2_ adenosine receptor binding subunit by photoaffinity crosslinking. Proc Natl Acad Sci U S A.

[16] Moss SM, Jayasekara PS, Paoletta S, Gao ZG, Jacobson KA (2014). Structure-based design of reactive nucleosides for site-specific modification of the A_2A_ adenosine receptor. ACS Med Chem Lett.

[17] Niiya K, Jacobson KA, Silvia SK, Olsson RA (1993). Covalent binding of a selective agonist irreversibly activates guinea pig coronary artery A_2_ adenosine receptors. Naunyn Schmiedeberg's Arch Pharmacol.

[18] Ji XD, Gallo-Rodriguez C, Jacobson KA (1993). 8-(3-Isothiocyanatostyryl) caffeine is a selective, irreversible inhibitor of striatal A_2_-adenosine receptors. Drug Dev Res.

[19] Guo D, Xia LZ, van Veldhoven JPD, Hazeu M, Mocking T, Brussee J, IJzerman AP, Heitman LH (2014). Binding kinetics of ZM241385 derivatives at the human adenosine A_2A_ receptor. ChemMedChem.

[20] Guo D, Pan AC, Dror RO, Mocking T, Liu R, Heitman LH, Shaw DE, IJzerman AP (2016). Molecular basis of ligand dissociation from the adenosine A_2A_ receptor. Mol Pharmacol.

[21] Guo D, Mulder-Krieger T, IJzerman AP, Heitman LH (2012). Functional efficacy of adenosine A_2A_ receptor agonists is positively correlated to their receptor residence time. Brit J Pharmacol.

[22] Smith PK, Krohn RI, Hermanson GT, Mallia AK, Gartner FH, Provenzano MD, Fujimoto EK, Goeke NM, Olson BJ, Klenk DC (1985). Measurement of protein using Bicinchoninic acid. Anal Biochem.

[23] Xiao WB, Nishimoto H, Hong H, Kitaura J, Nunomura S, Maeda-Yamamoto M, Kawakami Y, Lowell CA, Ra CS, Kawakami T (2005). Positive and negative regulation of mast cell activation by Lyn via the fc epsilon RI. J Immunol.

[24] Bas DC, Rogers DM, Jensen JH (2008). Very fast prediction and rationalization of pKa values for protein-ligand complexes. Proteins.

[25] Zhu K, Borrelli KW, Greenwood JR, Day T, Abel R, Farid RS, Harder E (2014). Docking covalent inhibitors: a parameter free approach to pose prediction and scoring. J Chem Inf Model.

[26] Motulsky AG (1984). Genetic-engineering, medicine and medical genetics. Biomed Pharmacother.

[27] Motulsky HJ, Mahan LC (1984). The kinetics of competitive radioligand binding predicted by the law of mass-action. Mol Pharmacol.

[28] Copeland RA (2005). Evaluation of enzyme inhibitors in drug discovery. A guide for medicinal chemists and pharmacologists. Methods Biochem Anal.

[29] de Zwart M, Vollinga RC, Beukers MW, Sleegers DF, Kunzel JKVD, de Groote M, IJzerman AP (1999). Potent antagonists for the human adenosine A_2B_ receptor. Derivatives of the triazolotriazine adenosine receptor antagonist ZM241385 with high affinity. Drug Dev Res.

[30] Weichert D, Kruse AC, Manglik A, Hiller C, Zhang C, Hubner H, Kobilka BK, Gmeiner P (2014). Covalent agonists for studying G protein-coupled receptor activation. P Natl Acad Sci USA.

[31] Segala E, Guo D, Cheng RK, Bortolato A, Deflorian F, Dore AS, Errey JC, Heitman LH, IJzermna AP, Marshall FH, Cooke RM (2016). Controlling the dissociation of ligands from the adenosine A_2A_ receptor through modulation of salt bridge strength. J Med Chem.

[32] Li C, Xu W, Vadivel SK, Fan PS, Makriyannis A (2005). High affinity electrophilic and photoactivatable covalent endocannabinoid probes for the CB1 receptor. J Med Chem.

[33] Ogawa G, Tius MA, Zhou H, Nikas SP, Halikhedkar A, Mallipeddi S, Makriyannis A (2015). 3'-functionalized adamantyl cannabinoid receptor probes. J Med Chem.

[34] Kijima H, Isobe Y, Muramatsu M, Yokomori S, Suzuki M, Higuchi S (1998). Structure-activity characterization of an H_2_-receptor antagonist, 3-amino-4-[4-[4-(1-piperidinomethyl)-2-pyridyloxy]-cis-2-butenylamino]- 3-cyclobutene-1,2-dione hydrochloride (T-066), involved in the insurmountable antagonism against histamine-induced positive chronotropic action in guinea pig atria. Biochem Pharmacol.

[35] Nijmeijer S, Engelhardt H, Schultes S, van de Stolpe AC, Lusink V, de Graaf C, Wijtmans M, Haaksma EEJ, de Esch IJP, Stachurski K, Vischer HF, Leurs R (2013). Design and pharmacological characterization of VUF14480, a covalent partial agonist that interacts with cysteine 98(3.36) of the human histamine H-4 receptor. Brit J Pharmacol.

[36] Narayanan A, Jones LH (2015). Sulfonyl fluorides as privileged warheads in chemical biology. Chem Sci.

[37] Lorenzen A, Beukers MW, van der Graaf PH, Lang H, van Muijlwijk-Koezen J, de Groote M, Menge W, Schwabe U, IJzerman AP (2002). Modulation of agonist responses at the A(1) adenosine receptor by an irreversible antagonist, receptor-G protein uncoupling and by the G protein activation state. Biochem Pharmacol.

[38] Zarek PE, Huang CT, Lutz ER, Kowalski J, Horton MR, Lindens J, Drake CG, Powel JD (2008). A(2A) receptor signaling promotes peripheral tolerance by inducing T-cell anergy and the generation of adaptive regulatory T cells. Blood.

[39] Beavis PA, Divisekera U, Paget C, Chow MT, John LB, Devaud C, Dwyer K, Stagg J, Smyth MJ, Darcy PK (2013). Blockade of A_2A_ receptors potently suppresses the metastasis of CD73(+) tumors. P Natl Acad Sci USA.

[40] Leone RD, Lo YC, Powell JD (2015). A2aR antagonists: next generation checkpoint blockade for cancer immunotherapy. Comput Struct Biotec.

[41] Mahoney KM, Rennert PD, Freeman GJ (2015). Combination cancer immunotherapy and new immunomodulatory targets. Nat Rev Drug Discov.

[42] Iannone R, Miele L, Maiolino P, Pinto A, Morello S (2014). Adenosine limits the therapeutic effectiveness of anti-CTLA4 mAb in a mouse melanoma model. Am J Cancer Res.

