# Supplementary material for: A covalent antagonist for the human adenosine A2A receptor
Source: Purinergic Signal. 2016 Dec 3;13(2):191–201. doi: 10.1007/s11302-016-9549-9 (PMC5432479; doi:10.1007/s11302-016-9549-9)
Supplement: Supplementary file 1 — (DOCX 122 kb) [file 11302_2016_9549_MOESM1_ESM.docx]

**Supporting information**

**A covalent antagonist for the human adenosine A_2A_ receptor**

Xue Yang, Guo Dong^1^, Thomas J.M. Michiels, Eelke B. Lenselink, Laura Heitman, Julien Louvel and Ad P. IJzerman*

Division of Medicinal Chemistry, Leiden Academic Centre for Drug Research, Leiden University, P.O. Box 9502, 2300 RA Leiden, the Netherlands

*Correspondence e-mail : ijzerman@lacdr.leidenuniv.nl

^1^Current address: Jiangsu Key Laboratory of New Drug Research and Clinical Pharmacy, Xuzhou Medical University, 209 Tongshan Road, Xuzhou 221004, Jiangsu, China.

**Chemistry pp 26-27**

**Biology pp 28-29**

**Chemistry**

All solvents and reagents were purchased from commercial sources and were of analytical grade. ^1^H NMR spectra were recorded on a Bruker AV 400 liquid spectrometer (^1^H NMR, 400 MHz) at ambient temperature. Chemical shifts are reported in parts per million (ppm), are designated by δ. Coupling-constants are reported in Hz and are designated as *J*. Analytical purity of the final compounds was determined by high pressure liquid chromatography (HPLC) with a Phenomenex Gemini 3μ C18 110A column (50 × 4.6 mm, 3 μm), measuring UV absorbance at 254 nm. Sample preparation and HPLC method was as follows: 0.5 mg of compound was dissolved in 1 mL of a 1:1:1 mixture of CH_3_CN/H_2_O/tBuOH and eluted from the column within 15 min, with a three component system of H_2_O/CH_3_CN/1% TFA in H_2_O, decreasing polarity of the solvent mixture in time from 80/10/10 to 0/90/10. All compounds showed a single peak at the designated retention time and are at least 95% pure. Liquid chromatography–mass spectrometry (LC–MS) analyses were performed using Thermo Finnigan Surveyor – LCQ Advantage Max LC-MS system and a Gemini C18 Phenomenex column (50 × 4.6 mm, 3 μm). The sample preparation was the same as for HPLC analysis. The elution method was set up as follows: 1–4 min isocratic system of H_2_O/CH_3_CN/1% TFA in H_2_O, 80:10:10, from the 4th min, a gradient was applied from 80:10:10 to 0:90:10 within 9 min, followed by 1 min of equilibration at 0:90:10 and 1 min at 80:10:10. Thin-layer chromatography (TLC) was routinely performed to monitor the progress of reactions, using aluminum coated Merck silica gel F254 plates. Purification by column chromatography was achieved by use of Grace Davison Davisil silica column material (LC60A 30–200 micron). Solutions were concentrated using a Heidolph laborota W8 2000 efficient rotary evaporation apparatus and by a high vacuum on a Binder APT line Vacuum Drying Oven. The procedure for the synthesis of similar compounds is given as a general procedure.

Scheme 1. Synthesis route of LUF7445.


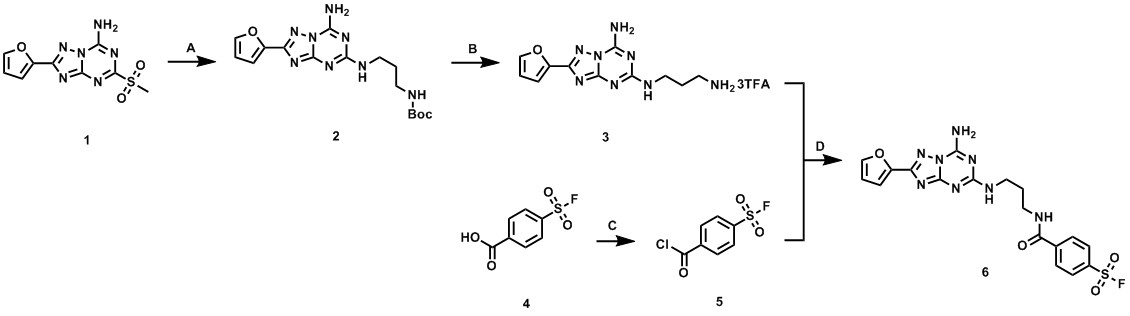


Reagents and conditions: A) *tert*-Butyl (3-aminopropyl)carbamate, MeCN, reflux, 60 h; B) TFA, DCM, room temperature, 0.5 h; C) SOCl_2_, reflux, 2 h ; D) triethylamine, dry dioxane, 70 °C, 18 h.

***tert*-Butyl (3-((7-amino-2-(furan-2-yl)-[1,2,4]triazolo[1,5-*a*][1,3,5]triazin-5-yl)amino)propyl)carbamate (2)**

2-(Furan-2-yl)-5-(methylsulfonyl)-[1,2,4]triazolo[1,5-a][1,3,5]triazin-7-amine **(1)** (280 mg, 1 mmol, 1 eq) , which was synthesized as previously reported [1], was suspended in acetonitrile (to give a 0.1 M solution). *tert*-Butyl (3-aminopropyl)carbamate (0.19 mL, 1.10 mmol, 1.1 eq) was added. The mixture was heated to 70 °C for 60 h. The final crude mixture was purified using flash column chromatography (DCM:EtOAc → EtOAc). Flash column (DCM:EtOAc, 50% → 100% EtOAc) gave a yellowish solid (310 mg, 0.83 mmol, 41% yield).**^1^H NMR** (400 MHz, DMSO-*d_6_*) δ 8.50–7.94 (m, 2H), 7.91–7.83 (m, 1H), 7.48–7.40 (m, rotamer 1, 0.30H), 7.36 (t, *J* = 5.6 Hz, rotamer 2, 0.70H), 7.04 (d, *J* = 3.3 Hz, 1H), 6.90–6.76 (m, 1H), 6.67 (dd, *J* = 3.0, 1.7 Hz, rotamer 3, 0.85H), 6.49–6.39 (m, rotamer 4, 0.15), 3.24 (q, *J* = 6.7 Hz, 2H), 2.97 (q, *J* = 6.4 Hz, 2H), 1.72–1.56 (m, 2H), 1.37 (s, 9H) ppm.

**N^5^-(3-aminopropyl)-2-(furan-2-yl)-[1,2,4]triazolo[1,5-*a*][1,3,5]triazine-5,7-diamine (3)**

Boc protecting groups were removed by dissolving **(2)** (374 mg, 1 mmol, 1 eq) in DCM, adding TFA (3.9 mL, 50 mmol, 50 eq) and removal of solvents after the reaction was finished. The remaining material was co-evaporated thrice with H_2_O and once with acetone, affording the triple TFA salt in quantitative yield. **^1^H NMR** (400 MHz, DMSO-*d_6_*) δ 8.61–8.04 (m, 2H), 7.88 (s, 1H), 7.70 (brs, 3H), 7.63-7.53 (m, 1H), 7.12–7.01 (m, 1H), 6.68 (dd, *J* = 3.3, 1.7 Hz, rotamer, 0.95H), 6.64–6.59 (m, rotamer, 0.05H), 3.40–3.28 (m, 2H), 2.85 (q, *J* = 6.7 Hz, 2H), 1.89–1.68 (m, 2H) ppm.

**4-Fluorosulfonyl benzoylchloride (5)**

Commercially available 4-fluorosulfonylbenzoic acid (500 mg, 2.45 mmo, 1 eql) was dissolved in thionyl chloride (8.89 mL, 122.4 mmol, 50 eq) and heated at 70 °C under N_2_ atmosphere. After 2 h, all material was dissolved and the thionyl chloride was removed by evaporation. The crude product was co-evaporated twice with toluene to give a brown liquid that solidified when it cooled down to room temperature. The resulting product was used directly in the next step. **^1^H NMR** (400 MHz, Acetone-*d_6_*) δ 8.52 (d, *J* = 8.5 Hz, 2H), 8.39 (d, *J* = 8.7 Hz, 2H).

**4-((3-((7-amino-2-(furan-2-yl)-[1,2,4]triazolo[1,5-*a*][1,3,5]triazin-5-yl)amino)propyl)carbamoyl) benzene sulfonyl fluoride (6) LUF7445**

**(3)** (TFA salt, 322 mg, 0.83 mmol, 1 eq) was added to dry dioxane (5 mL). **(5)** (184 mg, 0.92 mmol, 1.1 eq) was added along with triethylamine (0.3 mL, 2.49 mmol, 3 eq). The mixture was heated to 70 °C for 18 h. The reaction was finished, solvents were removed and a flash column (EtOAc→ 99% EtOAc + 1% AcOH) was used. Subsequent washing with acetone of the obtained solids gave the title compound as a white solid (77mg, 0.18 mmol, 22% yield).**^1^H NMR** (400 MHz, DMSO-*d_6_*) δ 8.90 (t, *J* = 6.0 Hz, 1H), 8.52–8.02 (m, 6H), 7.87 (s, 1H), 7.58–7.44 (m, 1H), 7.04 (d, *J* = 3.3 Hz, 1H), 6.68 (dd, *J* = 3.3, 1.8 Hz, 1H), 3.36-3.33 (m, 4H), 1.80 (pent, *J* = 6.3 Hz, 2H) ppm. **HPLC:** 96.7%, RT 7.40 min. **LC-MS**: [ESI+H]^+^: 461.13

**Reference**:

1. Guo D, Xia LZ, van Veldhoven JPD, Hazeu M, Mocking T, Brussee J, IJzerman AP, Heitman LH (2014) Binding Kinetics of ZM241385 Derivatives at the Human Adenosine A(2A) Receptor. Chemmedchem 9 (4):752-761. doi:10.1002/cmdc.201300474

**Biology.**

Table S1. Affinities of LUF7445 at the other adenosine receptor subtypes. Data are expressed as means ± SEM or percentage displacement at 1 μM of three separate experiments each performed in duplicate.

| **Compound** | *pK*_i_ or displacement at 1 μM (%)  hA_1_^a^ hA_2A_^b^ hA_2B_^c^ hA_3_^d^ | | | |
| --- | --- | --- | --- | --- |
| LUF7445 | 6.43 ± 0.095 | 8.99 ± 0.008 | 0.0% (2.0, -4.7,-2.6) | 7.31 ± 0.083 |

^a^Affinity determined from displacement of specific [^3^H]DPCPX binding on CHO cell membranes stably expressing human adenosine A_1_ receptors at 25^o^C during 3h incubation;

^b^ Affinity determined from displacement of specific [^3^H]ZM241385 binding from the hA_2A_AR at 25^o^C during 3h incubation;

^c^% displacement at 1 μM concentrations of specific [^3^H]PSB603 binding on CHO cell membranes stably expressing human adenosine A_2B_ receptors at 25^o^C during 3h incubation;

^d^Affinity determined from displacement of specific [^3^H]PSB-11 binding on CHO cell membranes stably expressing human adenosine A_3_ receptors at 25^o^C during 3h incubation.


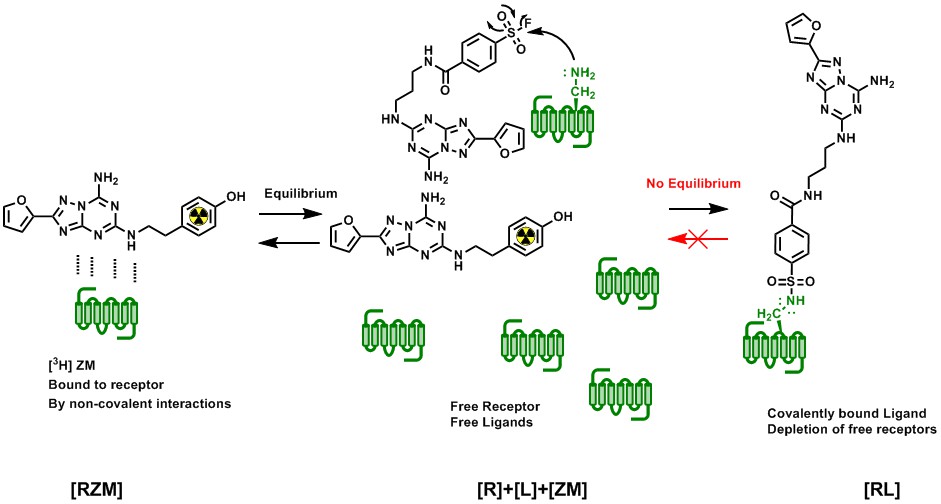


Scheme 2. Simplified representation of interaction between LUF7445 and hA_2A_AR during a competition association assay. The radiolabeled ZM-receptor complex ([RZM]) is being measured. If a compound L, such as LUF7445, binds covalently, the equilibrium shifts to the right ([RL]). The longer the incubation, the more receptors will be occupied by LUF7445.
